# Migration and Enrichment of Arsenic in the Rock-Soil-Crop Plant System in Areas Covered with Black Shale, Korea

**DOI:** 10.1100/tsw.2003.19

**Published:** 2003-04-07

**Authors:** Ji-Min Yi, Hyo-Taek Chon, Min Park

**Affiliations:** School of Civil, Urban and Geosystem Engineering, College of Engineering, Seoul National University, Seoul 151-744, Korea

**Keywords:** Okchon black shale, rock-soil-crop plant system, arsenic, biological absorption coefficient (BAC)

## Abstract

The Okchon black shale, which is part of the Guryongsan Formation or the Changri Formation of Cambro-Ordovician age in Korea provides a typical example of natural geological materials enriched with potentially toxic elements such as U, V, Mo, As, Se, Cd, and Zn. In this study, the Dukpyung and the Chubu areas were selected to investigate the migration and enrichment of As and other toxic elements in soils and crop plants in areas covered with black shale. Rock and soil samples digested in 4-acid solution (HCl+HNO_3_+HF+HClO_4_) were analyzed for As and other heavy metals by ICP-AES and ICP-MS, and plant samples by INAA. Mean concentration of As in Okchon black shale is higher than those of both world average values of shale and black shale. Especially high concentration of 23.2 mg As kg is found in black shale from the Dukpyung area. Mean concentration of As is highly elevated in agricultural soils from the Dukpyung (28.2 mg kg-1) and the Chubu areas (32.6 mg kg). As is highly elevated in rice leaves from the Dukpyung (1.14 mg kg) and the Chubu areas (1.35 mg kg). The biological absorption coefficient (BAC) of As in plant species decreases in the order of rice leaves > corn leaves > red pepper = soybean leaves = sesame leaves > corn stalks > corn grains. This indicates that leafy plants tend to accumulate As from soil to a greater degree than cereal products such as grains.

## INTRODUCTION

The increase in the number of heavy metal–emitting sources has aroused concern, and the degree and extent of these metal pollutions have been one of the main topics studied in environmental science[1]. The high levels of potentially toxic elements in black shale are of significance in environmental geochemistry. In soil systems, one of the main natural origins of As contamination is the black shale bedrock geology and many studies have shown the high levels of As and other potential toxic elements of concern to human and animal health[2,3,4]. Soil derived from these parent materials tend to reflect their extreme geochemical composition[5] and may influence human health by determining the elemental composition of crop plants[6].

The Okchon black shale in Korea provides a typical example of natural geological materials enriched with potentially toxic elements such as U, V, Mo, As, Se, Cd, and Zn[7]. The Okchon black shale is a member of the assemblages of strata, which is part of the Guryongsan Formation or the Changri Formation of Cambro-Ordovician age, which are parts of the Okchon Group in the central part of the southern Korean Peninsula[8]. The As enrichment of the Okchon black shale is reported by many studies[9,10,11,12]. Kim[13] has suggested that high levels of toxic elements in black shale may influence those of soils and crops in the Dukpyung area.

In this study the Dukpyung and the Chubu areas were selected, firstly, to investigate the concentration level of As and other toxic elements in soils and crop plants in areas covered with black shale; secondly, to evaluate the enrichment level of As in soils derived from black shale; and thirdly, to examine the uptake ratio of As from soils into crop plants.

## LOCAL GEOLOGY

The Okchon Zone has an average width of 80 km and shows a northeastern trend in the central part of Korea Peninsula. The Palaeozoic formation and the narrow Mesozoic formation, which are comprised mainly of limestone and clastic sedimentary rocks, are located in the northeastern part of the Okchon Zone. The Okchon Group located from the center to the southwest of Okchon Zone consists of metasedimentary rocks such as black shale, slate, grey chlorite schist, phyllite, hornblendite, and mica schist. The age of the Okchon Group has been the subject of debate for several decades. The two major thoughts about the age of the Okchon Group, which persist up to the present, are of Precambrian and Palaeozoic age. After a synthesizing study on the Okchon Group by Lee[14], he divided the group into two subgroups and proposed the ages of the Okchon Group to be Cambrian to early Ordovician and middle to late Ordovician, respectively.

Black shale in the Okchon Zone occurs in the Guryongsan Formation[8]. The Guryongsan Formation is also named as the Changri Formation in the southern part of the Okchon Zone. The study areas were selected within the zones of the Guryongsan Formation in Dukpyng area, and the Changri Formation in Chubu area (Fig. 1). Black shale of the Guryongsan Formation includes the uranium-bearing low graded coal seams in Dukpyung area. The Changri Formation consists mainly of schist, phyllite, black shale and slate, which include the narrow intercalated coal seam with 2 to 3 m width and uranium-enriched graphitic degradation coal.

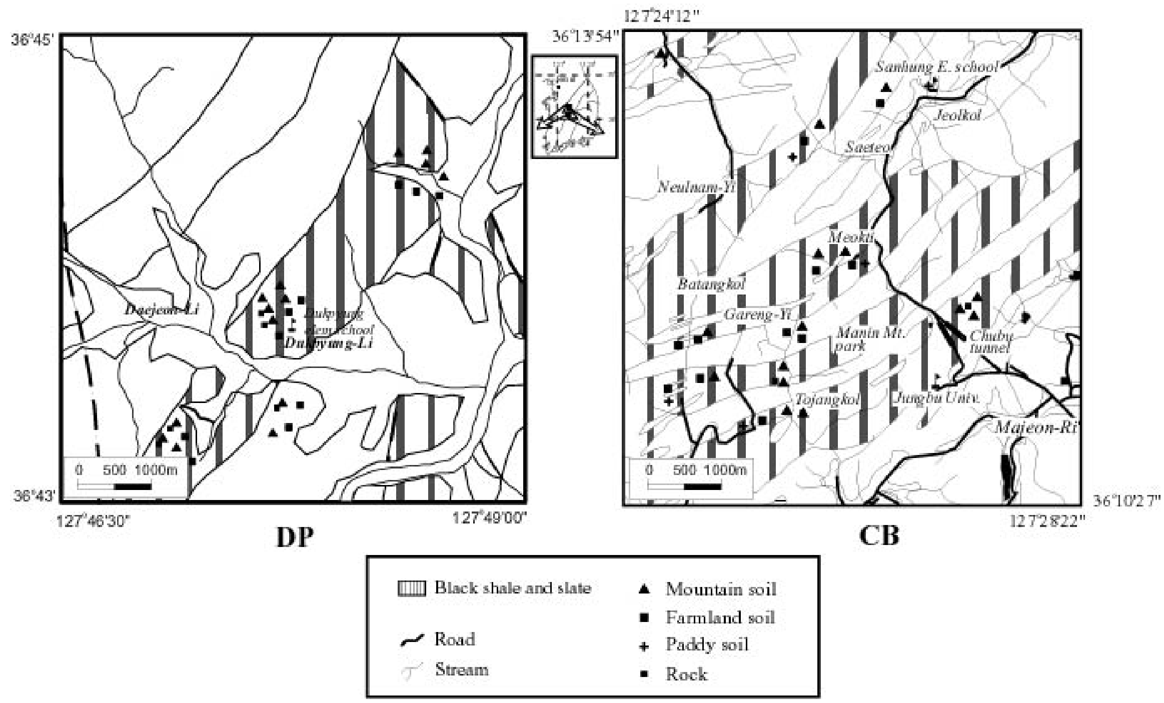

**FIGURE 1**. Geologic map and sampling localities of in the Dukpyung (DP) and Chubu (CB) areas in the Okchon zone. Lined parts indicate either the Guryongsan (DP) and Changri Formation (CB) which mainly consist of black shales and slates.

## MATERIALS AND METHODS

Black shale in the Guryongsan and the Changri Formations of the Okchon zone were collected from the available outcrops in the Dukpyung and the Chubu areas (Fig. 1). Rock samples were crushed to –10 mesh (<2 mm) in a jaw crusher, quartered, pulverized in a porcelain motar to –200 mesh (<80 μm), rehomogenized, and repackaged in sealed plastic bags. Surface soil samples (0- to 15 cm depth) were taken in the areas of black shale bedrock (Fig. 1) and classified into mountain, farmland, and paddy soils. Each soil comprised a composite of 15–20 subsamples taken across a 1- ×1- m square. Soil samples were disaggregated, sieved to –10 mesh (<2 mm), quartered, and pulverized into to –80 mesh (<180 μm) for chemical analysis. Rock and soil samples were analyzed in the Activation Laboratories Ltd. (ACTLAB) in Canada for As and other toxic elements by ICP-AES, ICP-MS and INAA. The samples were digested by using 4-acid (HCl + $HNO_3$ + HF + $HClO_4$), and were determined by ICP-AES and ICP-MS. However, volatile elements such as Hg, Se, and As were determined by INAA.

Crop plant samples such as rice, corn (leaves, stalks, grains), sesame, red pepper, and soybean leaves were collected to evaluate the uptake ratio of As from soils into plants (Fig. 1). Plants samples were vigorously washed in deionized water in order to remove the majority of particles of soil or dust adhering to the outside of plants, and then air-dried on covered trays in a filtered-air-drying cabinet at 40°C to prevent volatilization for 2 weeks. The dry plant samples were macerated and repackaged in sealed plastic bags. Plant samples were also analyzed in the ACTLAB for As and other toxic elements by ICP-MS. Plant samples was ashed at 480°C, and then digested with nitric acid. The procedure of sample preparation and chemical analysis is shown in Fig. 2.

A rigorous quality control program was implemented, which included reagent blanks, duplicate samples inhouse reference materials and certified international reference materials [23]. The precision and bias of the chemical analysis was less than 10%.

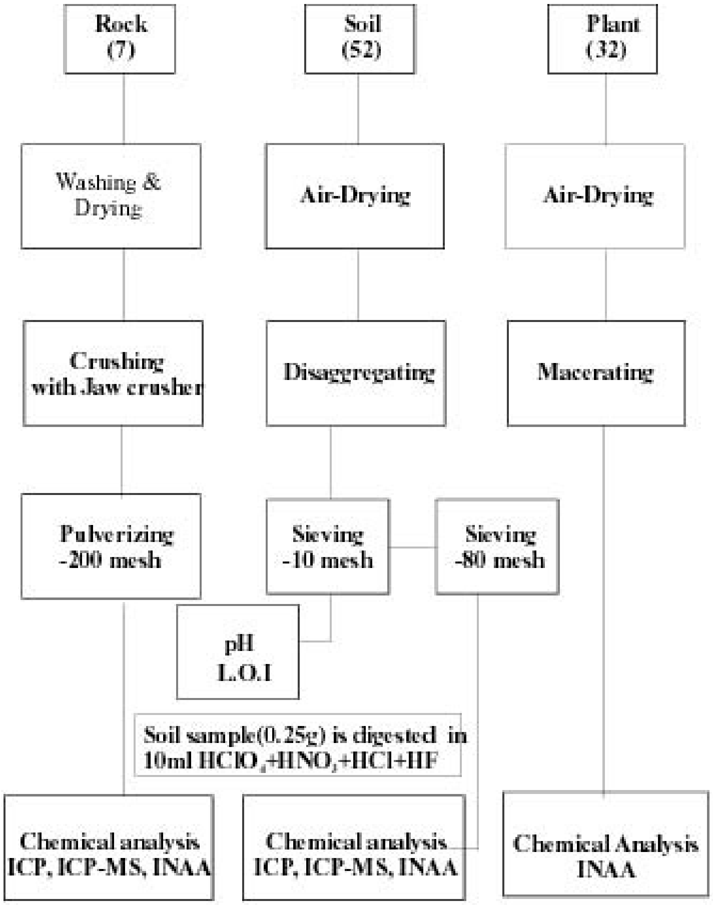

**FIGURE 2.** Flow chart showing the procedure of sample preparation and chemical analysis (figures in parenthesis are the number of samples).

## RESULTS AND DISCUSSION

### Geochemical Characteristics of Rocks and Soils

Range and mean concentrations of As and other heavy metals in black shale in the Dukpyung and the Chubu areas are shown in Table 1. Trace elements such as U, Ba, Mo, V, and Zn in black shale from the Chubu area show extremely higher concentration than those from the Dukpung area, which coincide with the result reported by Kim et al.[10] Uranium shows high correlation with Mo, Cd, Ni, Pb, Ba, Zn, and V in black shale from the Chubu area. However, mean concentrations of As, Ba, Cr, Mo, Ni, Se, Th, U, and V in black shale both from the Dukpyung and the Chubu area are much higher than those of world average shale[15] and average black shale[3]. Especially highest concentration of 28.2 mg As $kg^{-1}$ is found in black shale from the Dukpyung area, and 104 mg As $kg^{-1}$ from the Chubu area. Black shale in the Dukpyung and the Chubu areas may act as the sources of As and other toxic elements in the rock-soil system.

**Table 1**

**Range and mean concentrations of trace element in black shales from the study areas(unit in ppm).**

| Sample | | As | Ba | Cd | Cr | Cu | Mn | Mo | Ni | Pb | Se | Th | U | V | Zn |
|---|---|---|---|---|---|---|---|---|---|---|---|---|---|---|---|
| Dukpyung (n=4)[a] | Range | 10.8-28.2 | 86-20000 | 0.2-1.3 | 35-276 | 23-45 | 44-1948 | 1.7-20.1 | 36921 | 12-45 | 1.6-2.3 | 1.2-21.5 | 4.3-59.6 | 256-805 | 54-117 |
| Chu-bu (n=2) | Range | 9.6-104 | 860-19000 | 0.8 | 137-237 | 17-453 | 151-180 | 6.1-595 | 5-172 | 28-52 | 1.5-33.9 | 9.7-24.5 | 6.8-589 | 185-2356 | 75-135 |
| Earth crust[b] | | 2 | 580 | 0.1 | 100 | 50 | 1000 | 2 | 75 | 150 | 0.075 | 10 | 2.5 | 150 | 80 |
| Avg. shale[c] | | 13 | 580 | - | 90 | 45 | 850 | 3 | 68 | 140 | 0.6 | 11[e] | 3.7 | 130 | 95 |
| Avg. BS[d] | | - | 300 | 1 | 100 | 70 | 150 | 10 | 50 | - | - | - | - | 150 | <300 |

a: number of samples
b: Rose *et al.*, 1979
c: Turekian and Wedepohl, 1961
d: Vine and Tourtelot, 1970
e: Govett, 1981

Range and mean concentrations of trace elements in various soils overlying black shale from the Dukpyung and Chubu areas are shown in Table 2. Soils derived from black shale show significantly higher mean concentrations of As, Ba, Cu, Mo, Ni, Se, Th, U, V, and Zn than those in normal soil reported by Bowen[16] and Shacklette and Boerngen[17]. In particular, most mean concentrations of trace elements in soils from the Dukpyung area are higher than those of the Chubu area. But As concentration in soils from the Dukpyung area is higher than that of the Chubu area. Mean Co concentration in soils of the study areas is lower than that in normal soil which is similar to the result from the other black shale and slate in the Okchon belt[11,12]. Mean concentrations of Mn in mountain soils are normal and Pb in farmland soils from the Dukpyung and Chubu areas are also higher than those in normal soils.

Mean concentration of 61.7 mg As $kg^{-1}$ is found in mountain soils from the Dukpyung area, and 42.8 mg As $kg^{-1}$ in the Chubu area. As concentration is highly elevated in farmland soils from the Dukpyung area (28.2 mg/kg) and the Chubu area (32.6 mg/kg). These levels of As in soils are higher than the permissible level suggested by Kloke[18]. The permissible level means the threshold of the element concentrations in soils above which crops produced are considered as unsafe for human health. Some mountain soil samples from the Chubu area, which show significantly enriched level of trace elements, may be possibly contaminated due to uranium mining activity[10]. As shows significant correlations with Mo, Se, U, Cu, Sb, and Tl (Fig. 3). It has been suggested that these elements in the soils may be derived from the weathering of black shale[12]. But As is less correlated with Ni, Pb, Th, and Zn.

## Geochemical Characteristics Arsenic in Crop Plants

Range and mean concentrations of As in crop plants grown on soils overlying black shales in the Dukpyung and the Chubu area are shown in Table 3. Mean concentration of As in plant species decreases in the order of rice leaves (1.28 mg $kg^{-1}$) > corn leaves (0.35 mg $kg^{-1}$) > soybean leaves (0.19 mg $kg^{-1}$) > sesame leaves (0.17 mg $kg^{-1}$) > corn stalk (0.08 mg $kg^{-1}$) > red pepper (0.04 mg

**Table 2**

**Range and median concentrations of trace elements in various soils overlying black shale from the study areas(unit in mg/kg).**

| | Dukpyung | | Chubu | | | Normal soil[c] |
|---|---|---|---|---|---|---|
| | Mountain (n=10)[a] | Farmland (n=7) | Mountain (n=14) | Farmland (n=9) | Paddy (n=4) | |
| As | 39.8 | 26.8 | 30.4 | 23.3 | 21.5 | 6 |
| | (26.3-187)[b] | (11.5-52.7) | (5.8-123) | (16.3-114) | (11.1-39.2) | |
| Ba | 2950 | 1700 | 875 | 1000 | 1040 | |
| | (1200-10000) | (730-3800) | (560-37000) | (890-2500) | (910-2400) | |
| Cd | 0.7 | 0.5 | 0.3 | 0.3 | 0.7 | 0.35 |
| | (0.3-2.4) | (0.2-1.8) | (<0.1-5.7) | (0.2-1.8) | (0.3-1.7) | |
| Co | 21 | 24 | 25 | 21 | 21 | 30 |
| | (15-32) | (19-30) | (10-55) | (16-27) | (15-26) | |
| Cr | 142 | 139 | 113 | 107 | 114 | |
| | (92-171) | (114-180) | (92-205) | (68-130) | (98-241) | |
| Cu | 92 | 62 | 54 | 51 | 52 | 30 |
| | (59-238) | (59-74) | (33-320) | (33-63) | (49-55) | |
| Mn | 336 | 589 | 799 | 617 | 528 | 550[d] |
| | (180-756) | (339-1057) | (420-2136) | (407-1377) | (365-712) | |
| Mo | 35 | 12.7 | 5.3 | 3.9 | 4.3 | 1.2 |
| | (9.1-139) | (7.64-24) | (1.3-452) | (1.6-13.4) | (3.3-10) | |
| Ni | 73 | 61 | 56 | 53 | 57 | 30 |
| | (42-168) | (57-84) | (27-292) | (39.9-138.1) | (50-147) | |
| Pb | 49 | 27 | 30 | 30 | 31 | 35 |
| | (26-162) | (18-39) | (18-96) | (19-45) | (27-94) | |
| Se | 3.3 | 1.5 | 1.7 | 1.5 | 1.4 | 0.39[d] |
| | (1.7-10.2) | (0.7-3.5) | (1.3-9.0) | (0.5-2.4) | (1.1-2.1) | |
| Th | 20 | 17 | 18.7 | 17.2 | 16.6 | 9 |
| | (15.8-22.9) | (15.1-18.3) | (14.3-23.9) | (14.1-19) | (14.8-17.5) | |
| U | 33 | 14.3 | 6.5 | 6.4 | 7.9 | 2 |
| | (6-302) | (4.5-24.4) | (3.8-1160) | (4.3-21.3) | (5.7-18) | |
| V | 434 | 251 | 174 | 172 | 173 | 80[d] |
| | (234-2213) | (137-418) | (123-2407) | (125-373) | (140-307) | |
| Zn | 159 | 148 | 127 | 118 | 172 | 90 |
| | (95-353) | (115-217) | (74-120) | (85-267) | (112-272) | |

a : number of samples
b : range of elements concentrations
c : Bowen(1979)
d : Shacklette and Boerngen(1984)

kg$^{-1}$) = corn grain (0.04 mg kg$^{-1}$). Especially, As is highly elevated in rice leaves from the Dukpyung area (1.14 mg kg$^{-1}$) and the Chubu area (1.35 mg kg$^{-1}$). As level in rice leaves is much higher than the rice leaves (0.4 mg kg$^{-1}$) of the control area in the Dukpyung area, and also higher than the average concentration in rice[19] and the upper limit (0.20 mg kg$^{-1}$) of normal plants[20] (refer to Table 4). Relationship between As concentration in soils and crop plants is approximately positively correlated (Fig. 4).

In order to investigate the uptake of trace elements by plants, the biological absorption coefficient (BAC) is used in this study. The BAC of As in plant species decreases in the order of rice leaves (0.081) > corn leaves (0.013) > soybean leaves (0.006) = sesame leaves (0.006) > red pepper (0.055) > corn stalk (0.002) > corn grain (0.001) (see Table 3 and Fig. 7). This indicates that leafy plants tend to accumulate arsenic from soil to a greater degree than cereal products such as grain[21]. The BAC of arsenic according to the organs of corn shows decreasing tendency in the order of corn leaves > corn stalks > corn grains (Fig. 5). The relationship between the BAC of As in rice and As concentration in soil from the study areas shows roughly negative correlation (Fig. 6).

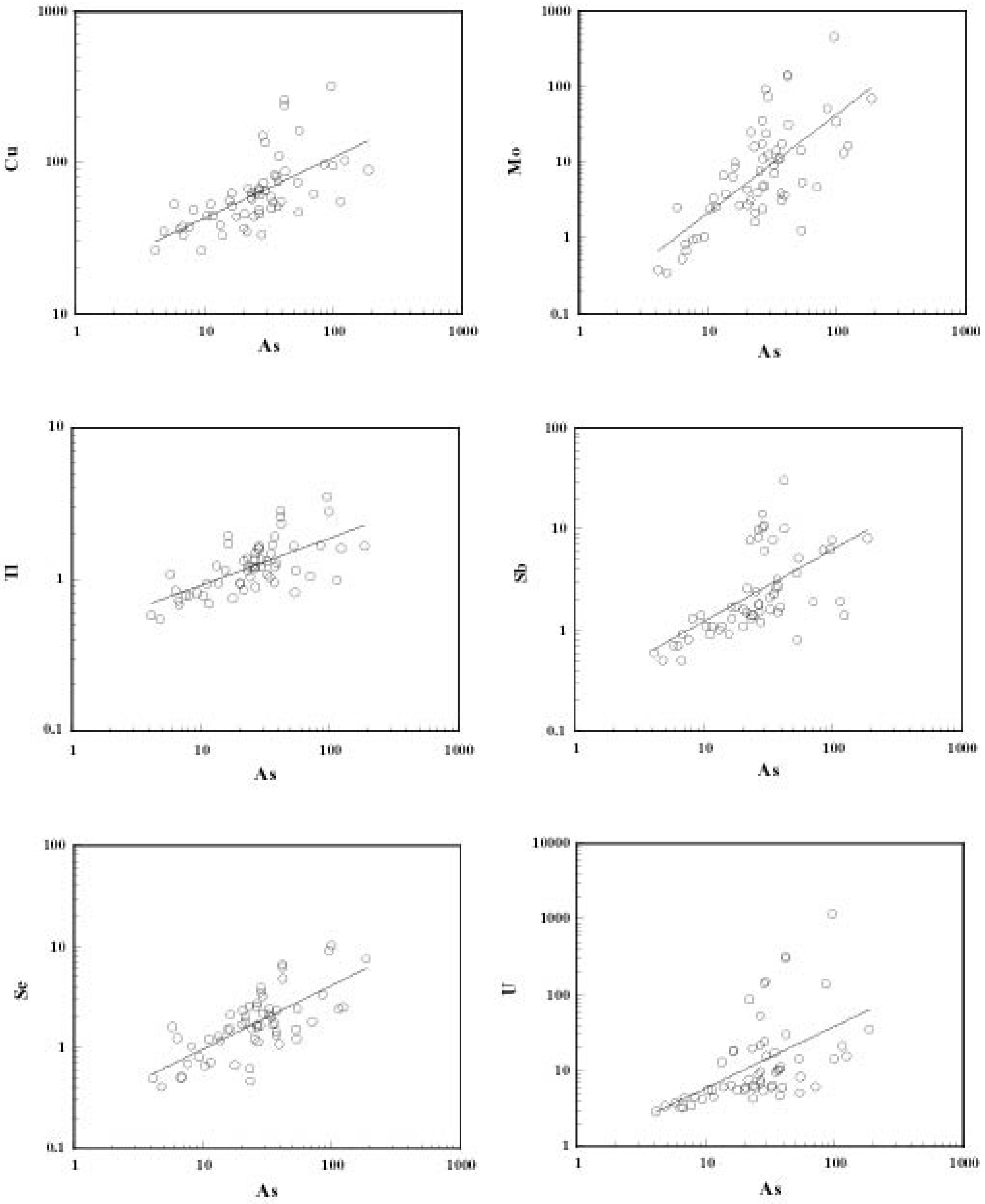

**FIGURE 3**. The As vs. Cu, Mo, U, Se, Sb, and Tl correlations of soils from the study areas.

**Table 3**
**Concentrations of As and BAC values of the crop plants from the study areas.**

| Location | Crop Plants | | As concentration (unit in mg/kg dry weight) | | BAC (mean) |
|---|---|---|---|---|---|
| Dukpyung | Corn | Leaves (n=3)[a] | Range | 0.12-0.35 | 0.008 |
| | | | Mean | 0.23 | |
| | | Stalks (n=1) | | 0.08 | 0.002 |
| | Rice | Leaves (n=6) | Range | 0.94-1.70 | 0.101 |
| | | | Mean | 1.14 | |
| | Red pepper | (n=2) | Range | 0.02-0.06 | 0.010 |
| | | | Mean | 0.04 | |
| | Sesame | Leaves (n=2) | Range | 0.12-0.16 | 0.006 |
| | | | Mean | 0.14 | |
| Chubu | Corn | Leaves (n=3) | Range | 0.23-0.83 | 0.017 |
| | | | Mean | 0.47 | |
| | | Grains (n=3) | Range | 0.03-0.04 | 0.001 |
| | | | Mean | 0.04 | |
| | Rice | Leaves (n=2) | Range | 1.2-1.5 | 0.060 |
| | | | Mean | 1.35 | |
| | Red pepper | (n=1) | | 0.04 | 0.001 |
| | Soybean | Leaves (n=2) | Range | 0.14-0.23 | 0.006 |
| | | | Mean | 0.19 | |
| | Sesame | Leaves (n=1) | | 0.19 | 0.006 |

a: number of samples

**Table 4**
**Arsenic concentration in the normal rice grain and normal plants (unit in mg/kg)**

| Element | Normal rice grain | | Normal Plants (Kabata-Pendias and Pendias. 1984) |
|---|---|---|---|
| | Rhu et al., 1988 | Kabata-Pendias and Pendias. 1984 | |
| As | 0.088 (0.02 - 0.15) | 0.11 - 0.20 | 1 - 1.7 |

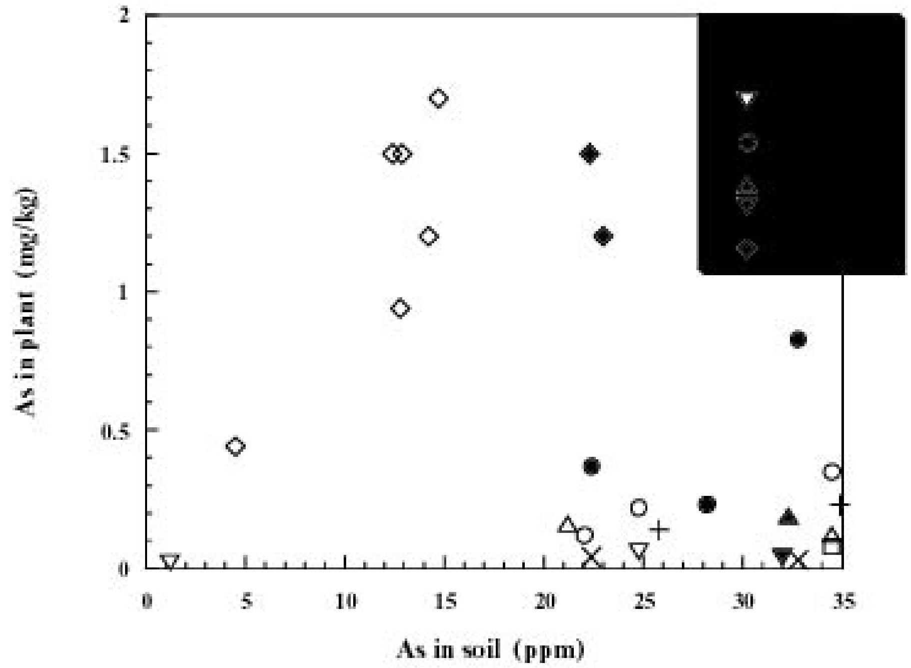

**FIGURE 4.** As concentrations of crop plants and soils from the study areas (crop plants from the Dukpyung area in open symbols and the Chubu area in closed symbols).

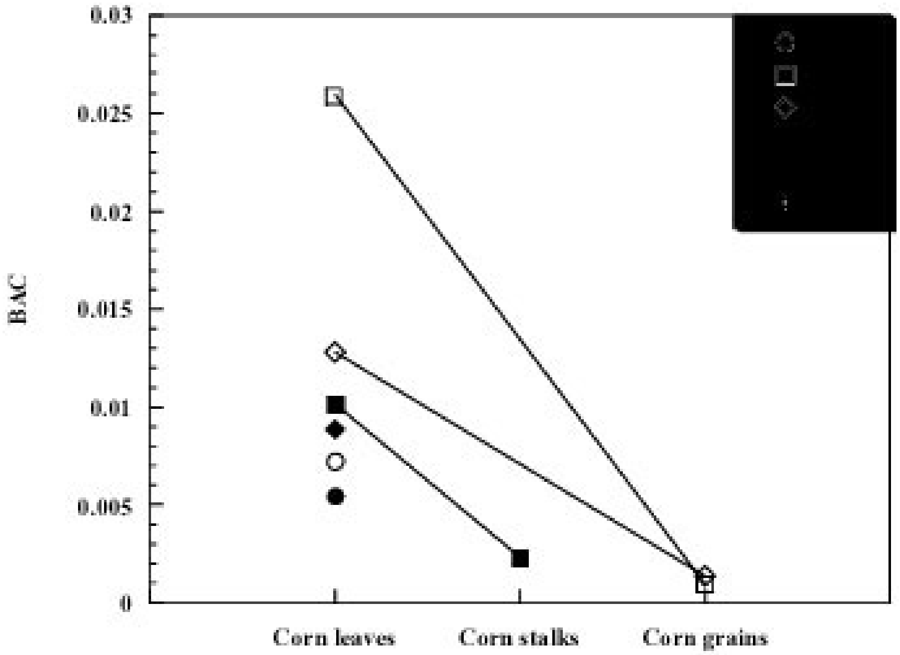

**FIGURE 5**. Biological absorption coefficient (BAC = Cp/Cs) of corn leaves, stalks, and grains from the study areas.

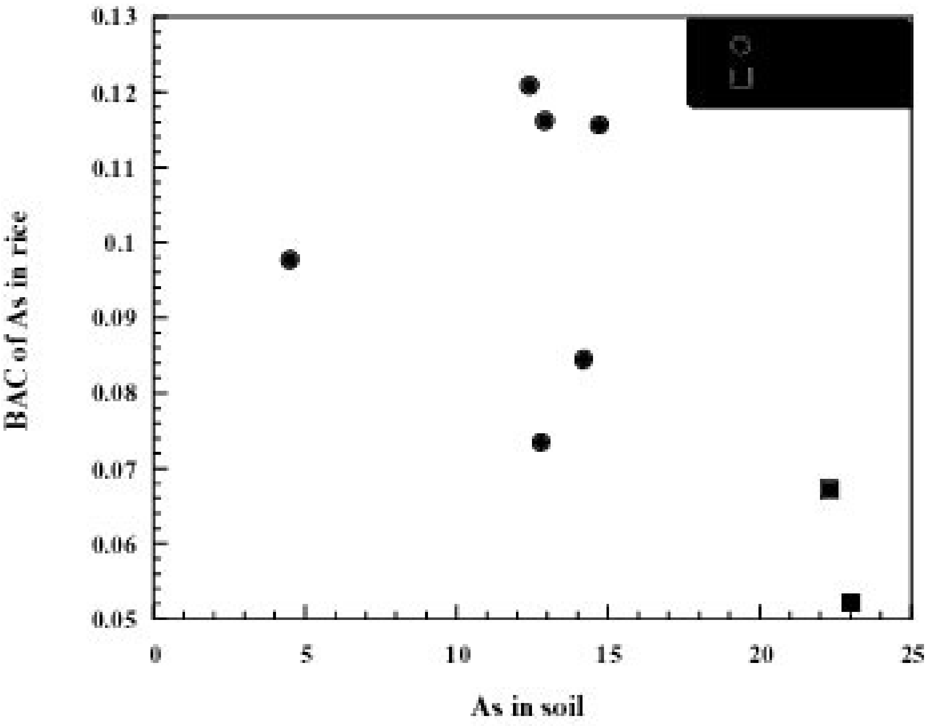

**FIGURE 6**. Biological absorption coefficient (BAC = Cp/Cs) of rice and As concentration in soil from the study areas.

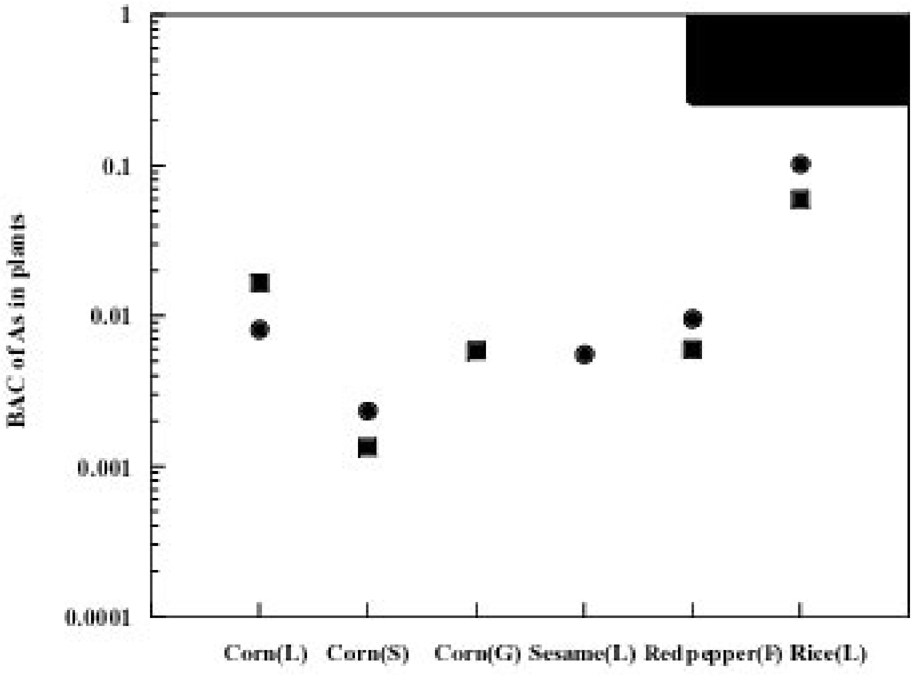

**FIGURE 7.** The variation of BAC in crop plants from the study areas (L: leaves, S: stalks, G: grains, and F: fruits).

## CONCLUSIONS

Arsenic and other toxic elements were enriched in the Okchon black shale. In particular, mean concentration of 23.2 mg As $kg^{-1}$ was found in black shale from the Dukpyung area and 56.8 mg As $kg^{-1}$ in the Chubu area. Geochemical properties of there bedrock may act as the sources of As to the overlying various soils. Mean concentrations of As in mountain soils from the Dukpyung and the Chubu areas are 61.7 and 42.8 mg $kg^{-1}$, respectively. As concentration was highly elevated in agricultural soils from the Dukpyung area (28.2 mg $kg^{-1}$) and the Chubu area (28 mg $kg^{-1}$). These levels of As in soils were higher than the permissible level in soil. As shows significant correlations with Mo, Se, U, Cu, Sb, and Tl. As is highly elevated in rice leaves from the Dukpyung area (1.14 mg $kg^{-1}$) and the Chubu area (1.35 mg $kg^{-1}$). The biological absorption coefficient (BAC) of As in plant species decreases in the order of rice leaves > corn leaves > soybean leaves = sesame leaves > red pepper > corn stalk > corn grain. The uptake degree of As by plants is more predominant in leaves than the other plant organs such as grains, which means that the foliage plants could behave as a good accumulator of As.

## ACKNOWLEDGEMENT

This study was financially supported by Korea Research Foundation Project No. KRF-2001-015-EP0010.

## References

1. Plants, J. and Raiswell, R. (1983) Principles of environmental geochemistry. In *Applied Environmental Geochemistry*. Thornton, I., Ed. Academic Press, London. pp. 1–39.
2. Armands, G. (1972) Geochemical studies of uranium and vanadium in a Swedish alum shale. *Stockholm Contrib. Geol*. 7, 1–148.
3. Vine, J.D. and Tourtelot, E.B. (1970) Geochemistry of black shale deposits - a summary report. *Econ. Geol.* 65, 253–272.
4. Kim, J.H. (1989) Geochemistry and genesis of the Guryongsan(Ogcheon) uraniferous black slates. *J. Korean Inst. Min. Geol*. 22, 35–63.
5. Bowie, S.H.U. and Thornton, I. (1984) *Environmental Geochemistry and Health*. Reidel, Dordrecht, 140 p.
6. Foth, H.D. (1978) *Fundamentals of Soil Science*. 4th ed. John Wiley & Sons, New York.
7. Kim, K.W. and Thornton, I. (1993) Influence of Ordovician uraniferous black shales on the trace element composition of soils and food crops, Korea. *Appl. Geochem*. *Suppl.* 2, 249–255.
8. Lee, C.H. and Kim, J.H. (1972) Explanatory Text of the Geological Map of Goesan. Geological Society of Korea, Seoul.
9. Chon, H.T., Cho, C.H., Kim, K.W., and Moon, H.S. (1996) The occurrence and dispersion of potentially toxic element in areas covered with black shales and slate in koea. *Appl. Geochem.* 2, 69–76.
10. Kim, J.S., Chon, H.T. (1997) Enrichment and dispersion of the potentially toxic elements in areas underlain by black shales and slates in the Chubu area, Korea. *J. Econ. Environ. Geol*. 30(2), 89–103.
11. Lee, J.S., Chon, H.T., and Kim, K.W. (1998a) Migration and dispersion of trace elements in the rock-soil-plant system in areas underlain by black shales and slates of Okchon Zone, Korea. *J. Geochem. Explor*. 65, 61–78.
12. Lee, J.S., Chon, H.T., Kim, J.S., Kim, K.W., and Moon, H.S. (1998b) Enrichment of potentially toxic elements in areas underlain by black shales and slates in Korea. *Environ. Geochem. Health* 20, 135–147.
13. Kim, K.W. (1993) Influence of Uraniferous Black Shales on Trace Elements in Soils and Crops in Korea [Ph.D Thesis]. University of London, 410 pp.
14. Lee, D.J. (1986) Mineralogy of low-grade uranium ores in the black slate of the Ogcheon Group, Korea. *J. Korean Inst. Min. Geol*. 19, 133–146.
15. Turekian, K.K. and Wedepohl, K.H. (1961) Distribution of the elements in some major units of the earth's crust. *Geol. Soc. Am. Bull.* 72, 175–192.
16. Bowen, H.J.M. (1979) *Environmental Chemistry of the Elements*. Academic Press, London, 333 p.

17. Shacklette, H.T. and Boerngen, J.G. (1984) Element Concentrations in Soils and Other Surficial Materials of the Conterminous United States. USGS Prof. Paper 1270. U.S. Government Printing Office, Washington, D.C.
18. Kloke, A. (1979) Contents of Arsenic, Cadmium, Chromium, Fluorine, Lead, Mercury and Nickel in Plants Grown on Contaminated Soil. UN-ECE Symposium, Geneva.
19. Rhu, H,I., Suh, C.M., Jun, S.H., Lee, M.H., Yu, S.J., Huh, S.N., and Kim, S.Y. (1988) A Study on the Natural Content of Heavy Metals in Paddy Soil and Brown Rice in Korea. Report of NIER, Korea. Vol. 10. pp. 155–163.
20. Kabata-Pendias, A. and Pendias, H. (1984) *Trace Elements in Soils and Plants*. CRC Press, Boca Raton, FL, 315 p.
21. Adriano, D.C. (1986) *Trace Elements in the Terrestrial Environment*. Springer-Verlag, New York, 533 p.
22. Govett, G.J.S. (1981) *Rock Geochemistry in Mineral Exploration*. Elsevier, Amsterdam.
23. Ramsey, M.H., Thomson, M., and Banerjee, E.K. (1987) Realistic assessment of analytical data qualityfrom inductively coupled plasma atomic emission spectrometry. Anal. Proc. 24, 260-265.
